# Junge Menschen in der Politikberatung: Es braucht neue Infrastrukturen der Beteiligung für junge Menschen in politischen Beratungsprozessen

**DOI:** 10.1007/s12054-022-00537-5

**Published:** 2022-12-12

**Authors:** Nadia Kutscher, Wolfgang Schröer

**Affiliations:** 1 Köln, Deutschland; 2 Hildesheim, Deutschland

**Keywords:** Partizipation, Mitbestimmung, Kinder- und Jugendpolitik, Beratung, Politik, UN-Kinderrechtskonvention

## Abstract

Diese Einleitung in den Schwerpunkt „Junge Menschen, Politikberatung und Beteiligung“ skizziert kurz die Probleme, vor denen der Wunsch nach politischer Beteiligung von Kindern und Jugendlichen während der Covid-Pandemie stand und stellt dann die Beiträge des Schwerpunkts vor.

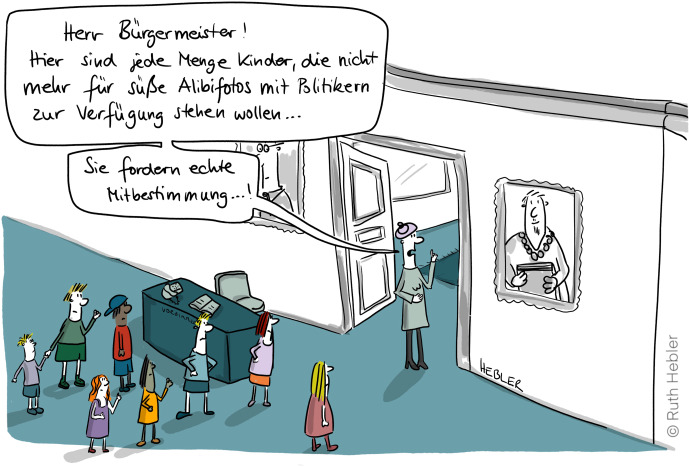



Die Beteiligung junger Menschen in politischen Gestaltungsprozessen ist zu einem Megathema der Kinder- und Jugendpolitik geworden. Kaum ein Tag vergeht, in dem nicht die Bedeutung von Partizipation und Mitbestimmung von Kindern und Jugendlichen in Fachzeitschriften gefordert und die fehlende Beteiligung junger Menschen problematisiert wird. Die Regulation der Corona-Krise hat diese Diskussion noch verstärkt.

Junge Menschen fühlten sich kaum gehört und sahen kaum Möglichkeiten, die politischen Regulationen der Covid-19-Pandemie mitzugestalten (Andresen et al. 2022). Und noch mehr: Auch in Belangen, die sie unmittelbar betreffen, wie die Corona-Politik im Bildungswesen, wurden zuerst die Fachkräfte, wie Lehrer_innen etc., dann Eltern und erst sehr spät und zuletzt die Schüler_innen und damit die jungen Menschen gehört.

Doch: Sollen Beteiligung, Partizipation und Mitbestimmung nicht zu allgemeinen Floskeln in der politischen Landschaft verkommen, die gut gemeint, aber in wenig nachhaltige Infrastrukturen eingebettet sind und nur einige Hochglanz-Broschüren hervorbringen, gilt es, sich differenziert mit unterschiedlichen Bedingungen in einzelnen Politikfeldern und Prozessen der Politikgestaltung auseinanderzusetzen. In diesem Zusammenhang ist der Blick in den letzten Jahren auch auf die Politikberatung gerichtet worden.

Politikberatung wird vor allem mit Fachverbänden, Lobbyist_innen und bezahlten Beratungsfirmen verbunden. Unterschiedliche Ministerien und Behörden haben diesbezüglich auch schon mit eigenen Merkwürdigkeiten im Einkauf von Beratungsdienstleistungen für Schlagzeilen gesorgt. Wenige Schlagzeilen haben bisher allerdings junge Menschen in politischen Beratungsprozessen machen können, denn sie werden weitgehend ausgegrenzt. In der Politikberatung finden sich in erster Linie ältere Erwachsene. Junge Menschen sind hier nur selten in den Beratungskommissionen. Sie kommen – sieht man einmal von kommunalen Jugendparlamenten ab – nicht in den etablierten politischen Beratungsprozessen von Bund und Ländern, sondern, wenn überhaupt, in kurzen eventartigen Anhörungen vor. Diese werden dann vor allem für die Öffentlichkeitsarbeit genutzt, ohne dass klar ist, wie die Ergebnisse der Anhörungen weiter aufgenommen werden.

Das Bundesjugendkuratorium hat 2019 in einer umfassenden Stellungnahme „Junge Menschen in der Politikberatung“ bereits herausgearbeitet, dass junge Menschen ein Recht haben, grundlegend in politische Beratungsprozesse einbezogen zu werden. Dieses Recht ist nicht nur durch die UN-Kinderrechtekonvention untersetzt, sondern dessen Verwirklichung auch eine Frage der Generationengerechtigkeit. In einer demokratischen Wissensgesellschaft ist es zudem von Bedeutung, wer wie in die politischen Wissensbildungsprozesse eingebunden wird, welche Beratungsinfrastrukturen der politischen Wissensbildung von politischen Stakeholdern und Entscheidungsträger_innen existieren und inwiefern bzw. wie junge Menschen darin angesprochen werden.

Gleichzeitig sind diese Entscheidungsträger_innen auch darauf angewiesen, sich immer wieder über den aktuellen Stand des Wissens aus ganz unterschiedlichen Perspektiven zu informieren. In diesem Zusammenhang ist es schlicht ein politischer Fehler, junge Menschen nicht systematisch in die politischen Beratungsprozesse zu integrieren, denn sie haben eine eigene politische Erfahrungsdimension, die sich auch aus der Machtasymmetrie zwischen Erwachsenen und jungen Menschen ergibt, aber auch aus ihrer anderen generationalen und zeitlichen Perspektive. Bestes Beispiel hierfür ist die Klimakrise.

Entsprechend verfügen sie über eigene Sichtweisen aus ihrem Alltag und ihren Lebenslagen auf Politik, die nicht von Erwachsenen repräsentiert werden können. Sie sind zudem Akteur_innen in Feldern, wie zum Beispiel in der Bildungsinfrastruktur oder in der Kinder- und Jugendhilfe, die nur für sie politisch gestaltet werden. Hier können sie vor allen Dingen Auskunft darüber geben, wie diese zukünftig weiterzuentwickeln wären und politisch gestaltet werden können.

Doch nicht nur in diesen Feldern, in denen unmittelbar die Lebenslage Kindheit und Jugend gestaltet wird, sind junge Menschen stärker in die politischen Beratungsprozesse einzubeziehen. Gerade auch in Zukunftsfragen, wie sie sich in der Finanz- und Haushaltspolitik oder in der Verteidigungspolitik und nicht zuletzt in der Klimapolitik stellen, sind die Positionen und Perspektiven junger Menschen nicht nur anzuhören, sondern es ist mit ihnen das zu berücksichtigende, relevante Wissen in der politischen Wissensbildung zu entwickeln. Es gibt keinen Grund anzunehmen, dass die politische Beratung durch junge Menschen aus zivilgesellschaftlichen Gruppen eine geringere Qualität (im Sinne einer geringeren Komplexität) hat als die der Erwachsenen.

Gleichzeitig streicht *Truc-Quynh Vo* in dem Interview „Einfach mal den Schalter der Erwachsenen umlegen“ in diesem Schwerpunkt heraus, dass ein großer Graben zwischen jungen Menschen und Erwachsenen entstanden ist, was Beteiligungsprozesse in der Politikberatung betrifft. Dieser Graben lässt sich nicht so einfach überspringen, sondern er erfordert die Gestaltung von Infrastrukturen, die erst einmal die Machtasymmetrie zwischen jungen Menschen und Erwachsenen regulieren können, um mehr als nur eine Alibi-Beteiligung in diesem Kontext zu ermöglichen. Deutlich wird darin auch, dass es schon Enttäuschungserfahrungen gibt, vor deren Hintergrund es erforderlich ist, verbindlich zu zeigen, dass jede Neueinladung zu Beteiligung und Beratung nicht wieder Alibifunktion hat, sondern wirklich ernst gemeint ist und Folgen nach sich zieht.

Darauf verweist auch der Beitrag von *Walburga Hirschbeck* in unserem Schwerpunkt. Sie macht explizit deutlich, dass es nicht nur entscheidend ist, junge Menschen aus ganz unterschiedlichen Lebenslagen in Beratungsprozesse einzubeziehen und damit das Nichtrepräsentiertsein relevanter Gruppierungen zunehmend zu überwinden. Sie weist auch darauf hin, dass es zwar viele Beteiligungsaktivitäten gibt, in denen Kinder und Jugendliche eingeladen sind, ihre Ideen und Wünsche zu äußern. Doch, wie auch schon das Bundesjugendkuratorium (2019) mahnt, findet in der Regel kein systematisches Monitoring statt, was aus diesen Anregungen wird und es wird auch kein Dialog hierzu mit den befragten jungen Menschen geführt. Vor diesem Hintergrund verwundert es nicht, wie es auch im Interview mit *Truc-Quynh Vo* anklingt, dass junge Menschen sich nicht ernst genommen fühlen und sich fragen, ob sie überhaupt noch an solchen Aktivitäten teilnehmen sollen.

Im Gespräch von *Yola Fanroth* (Schülerin), *Vincent Sipeer* (Student) mit *Holger Hofmann* vom Deutschen Kinderhilfswerk, das mit rund 100 weiteren Organisationen in der sogenannten National Coalition Deutschland, dem Netzwerk für die Umsetzung der UN-Kinderrechtskonvention, vertreten ist, wird deutlich, welche Chancen darin liegen, wenn Kinder und Jugendliche an der Politikberatung beteiligt werden. Die Gesprächspartner_innen benennen dabei deutlich, wie relevant diese wirklichen Beteiligungserfahrungen für die Einsozialisation in eine Demokratie sind. Dazu gehört auch, die jungen Menschen dabei zu begleiten und zu befähigen, ihre Anliegen in den politischen Willensbildungsprozessen wirkmächtig einzubringen.

Ein wichtiges demokratisches Mitwirkungsinstrument sind Wahlen. Seit einigen Jahren wird diskutiert, das Alter für das aktive Wahlrecht zu senken, um damit mehr jüngere Menschen politisch zu beteiligen. Getan hat sich in dieser Hinsicht lange Zeit nichts, wie *Wendelin Haag* in seinem Beitrag konstatiert und gleichzeitig erläutert, warum es wichtig wäre, Jugendlichen früher als bisher das Wahlrecht zuzugestehen – immerhin steht das Thema inzwischen im aktuellen Koalitionsvertrag, was hoffen lässt.

Wie aber sollen junge Menschen Demokratie als einen Zusammenhang, in dem sie relevant sind, in dem ihre Stimme Gehör findet und in dem sie und ihre Interessen vertreten werden, erfahren (und nicht nur die Anliegen der ressourcenreichen Akteur_innen), wenn sie immer wieder erleben, dass sie darin nicht vorkommen? Eine Gesellschaft, die nicht immer mehr junge Menschen erleben will, die öffentlichen Einrichtungen nicht zutrauen, dass dort ihre Themen, Ängste, Sorgen und Vorstellungen ernst genommen werden und weniger vertrauen und zunehmend ‚alternative Fakten‘ für real halten (vgl. Bepanthen-Kinderförderung 2022), kann sich nicht leisten, junge Menschen, um deren Zukunft es geht, nicht zu berücksichtigen. Die Studie verweist dabei auf einen deutlichen Zusammenhang zwischen sozioökonomischem Status des Elternhauses von Kindern und Jugendlichen und Vertrauen in öffentlichen Einrichtungen. Hier deutet sich an, dass die schon in der Studie der Friedrich-Ebert-Stiftung von 2006 thematisierte Erfahrung, nicht gehört zu werden, zu einer Distanzierung von staatlichen Strukturen und einer Verdrossenheit gegenüber etablierten Entscheidungsprozessen führen kann und dies insbesondere ressourcenbenachteiligte Gruppen, die auch in den Beteiligungsaktivitäten und erst recht in Politikberatungszusammenhängen unterrepräsentiert sind, betrifft.

Im Zusammenhang mit der Frage, wer in der Regel beteiligt wird, ist ein immer wieder ungeklärtes Thema, wie einerseits die Stimme der Kinder, Jugendlichen und jungen Erwachsenen innerhalb etablierter politischer Strukturen zu Gehör kommen kann, wenn diese Beteiligung innerhalb sehr spezifischer, oft formalisierter und sehr voraussetzungsvoller Prozesse stattfindet und andererseits dabei nicht nur „Beteiligungs-Profis“ wie Funktionär_innen aus der verbandlichen Arbeit, die i. d. R. nicht über die Erfahrungen ressourcenbenachteiligter junger Menschen verfügen und damit auch deren Perspektive nur eingeschränkt repräsentieren können, einbezogen werden sollen.

Kinder- und Jugendbeteiligung in der – dann oft kommunalen – Politikberatung verbleibt zumeist im Rahmen des Äußerns von Wünschen oder Projekte mit kleinem Budget in Bezug auf Spielplätze, Halfpipes o. ä. Zu anderen (‚Erwachsenen‘-) Themen werden sie, wie oben angemerkt, kaum befragt. Die große Herausforderung bleibt, Infrastrukturen zu schaffen, die eine Brücke zwischen dem etablierten Politiksystem und den Erfahrungen und dem Wissen der jungen Menschen in unterschiedlichen – ressourcenprivilegierten wie ressourcenbenachteiligten – Lebenslagen schlagen und dabei die Differenz an Erfahrungen und Fähigkeiten in Prozessen der Politikberatung ausgleichen.
